# Dr. Albert Curtis Purvil

**Published:** 1916-07

**Authors:** 


### OBITUARY

---

**Readers are requested to report promptly the death of all physicians in Western New York, or former residents of this region, or graduates of any medical school in Western New York, and to notify the families of the deceased of our desire to publish adequate Obituary notices.**

---

**Dr. Albert Curtis Purvil** (not listed in Polk nor State Directory), died at his home in Brooklyn, May 30 of sleeping sickness, contracted from a tsetse bite in the Belgian Congo, several years ago.

